# Does a low-carbohydrate diet impede endurance sports performance? Debate Consensus

**DOI:** 10.1016/j.ajcnut.2026.101271

**Published:** 2026-05-01

**Authors:** Louise M Burke, Timothy D Noakes

**Affiliations:** 1 Centre for Human Metabolism and Performance, Mary MacKillop Institute for Health Research, Australian Catholic University, Melbourne, Victoria, Australia; 2 University of Cape Town, Cape Town, South Africa

**Keywords:** keto-adaptation, carbohydrate availability, athletic performance, exercise carbohydrate intake, exercise hypoglycaemia

## Abstract

According to current scientific consensus, strategies that promote the availability of body carbohydrate (CHO) stores to meet the fuel needs of endurance sport represent the best approach to optimize performance. Such strategies are known to achieve a range of metabolic and nonmetabolic benefits during both high intensity exercise of short duration and more prolonged exercise of lower intensity. This debate considered a counter proposal to the concept that fat oxidation cannot supply ATP sufficiently rapidly to support performance in such events. Indeed, the “ketogenic low-CHO high-fat” diet can increase the athlete’s capacity to oxidize the more plentiful body fat stores. Even here, however, CHO ingestion during exercise improves performance of prolonged events by preventing exercise-induced hypoglycemia. This debate sought to advance current understanding of the competing ideas that to maximize either their CHO or fat oxidation during exercise and so optimize their performance, endurance athletes should eat diets rich in either CHO or fat. Points of agreement include the muscle’s capacity to be trained to increase oxidation of either fuel, whereas points of disagreement include whether strategies to promote highest rates of CHO oxidation provide advantages over the amounts needed to prevent hypoglycemia. Future research should target challenges in measuring both metabolism and performance.

The current global scientific consensus is that performance during both high intensity exercise of short duration and prolonged exercise of lower intensity is enhanced by strategies that achieve high carbohydrate (CHO) availability to optimize its use by the exercising muscles. This consensus is based on the range of attributes provided by CHO during exercise: an economical fuel source for the muscle and central nervous system with both oxygen-dependent and oxygen-independent pathways, and capacity for performance benefits from nonmetabolic mechanisms. CHO oxidation, initially from muscle glycogen and then, as progressive muscle glycogen depletion develops, from ingested (exogenous) CHO, is obligatory to maximize human exercise performance. It is also argued that, even though the mean exercise intensity [maximal aerobic capacity (%VO_2_max)] is lower in longer sporting events, ATP generation from fat oxidation is still inadequate to sustain an optimum exercise performance for all characteristics of the event. It is irrefutably true that the oxidation of CHO from muscle glycogen, supplemented with high rates of CHO oxidation from exogenous sources, produces a lower, more economical muscle oxidative substrate (ATP yield per oxygen consumed) than does fat oxidation. This may become important during higher intensity exercise (>80%VO_2_max), throughout, or at critical moments in a sporting event, when the athlete is already close to the speeds/power output that can be sustained for prolonged periods. In addition, CHO intake during exercise may produce other beneficial effects such as maintenance of normoglycemia and a “mouth-sensing” interaction with the central nervous system to reduce perceived effort.

Countering this argument are a number of recent publications measuring equivalent exercise performances in both high intensity exercise of short duration and more prolonged exercise at a lower intensity in the same subjects after they had eaten either low-CHO high-fat (LCHF) or high-CHO low-fat (HCLF) diets for ≥4 wk before exercise testing. These studies challenge the prevailing consensus that fat oxidation cannot supply ATP sufficiently rapidly to support either prolonged exercise or exercise of moderate to high intensity. Rather, the counter proposal is that these findings support the initial proposition that, by increasing the athlete’s capacity to oxidize the more plentiful body fat stores, the “ketogenic LCHF” diet would remove the reliance on muscle glycogen oxidation and its supplementation from high rates of exogenous CHO ingestion during endurance exercise. Although such studies have established that high rates of CHO oxidation from muscle glycogen and exogenous ingested CHO are not essential for endurance performance under all conditions, there is a clear consensus that CHO ingestion improves performance during prolonged exercise even in athletes adapted to the ketogenic LCHF diet. The argument presented here, which has yet to achieve consensus, is that this effect is due to the prevention of exercise-induced hypoglycemia when hepatic glucose production falls behind an inevitable and progressive increase in blood glucose oxidation during more prolonged exercise.

The debate allowed a high level of discussion and identified the key points of agreement (Table 1) and intellectual difference (Table 2) between the 2 participants. Noakes presented evidence from several randomized controlled trials (RCTs) showing equivalent exercise performance in subjects participating in events lasting from minutes to hours after they had eaten either the LCHF or the HCLF diet for ≥4 wk. Burke challenged this conclusion by presenting a dashboard of studies measuring performance with LCHF diets alone compared with those with higher CHO availability, ranging from chronic high CHO availability to CHO added to a chronic LCHF diet.

**TABLE 1:** Points of agreement

1	By maintaining euglycemia, CHO ingestion during prolonged exercise can improve performance.
2	Adaptation to an LCHF diet substantially increases rates of fat oxidation during exercise.
3	This adaptation can occur quite rapidly in elite athletes, within as few as 5–7 d.
4	High rates of fat oxidation can occur at exercise intensities of ≥65%VO_2_max in athletes adapted to an LCHF diet.
5	Adaptation to an LCHF diet increases the oxygen requirement for substrate oxidation due to the stoichiometry of fat vs. CHO oxidation, resulting in higher oxygen consumption during submaximal exercise for the same ATP yield (i.e., power or speed production).
5	Respiratory gas analysis is not ideal for the calculation of substrate oxidation during non–steady-state conditions or at exercise intensities that exceed the “lactate” or “anaerobic” thresholds.

Abbreviations: CHO, carbohydrate; LCHF, low-carbohydrate high-fat; VO_2_max, maximum aerobic capacity.

**TABLE 2:** Continuing controversies

1	Whether or not CHO availability during exercise, manipulated by a range of strategies including pre-exercise CHO intake and CHO intake during exercise, influences subsequent exercise performance via several independent and interactive mechanisms.
2	Whether the oxidation of CHO is more beneficial than fat oxidation because it can produce ATP via anaerobic pathways and its oxidation during exercise requires lower oxygen consumption (improved economy).
3	Whether performance during exercise of high intensity and prolonged duration is dependent on muscle glycogen or, when depletion occurs, it can only be maintained by high rates of exogenous CHO provision (up to 120 g/h) and oxidation.
4	Whether the localized depletion of muscle glycogen depletion can cause fatigue and/or reduce exercise capacity/performance during prolonged exercise.
5	Whether exercise-induced hypoglycemia, which can be eliminated by consuming small amounts of CHO (10 g/h) during exercise, is the sole determinant of performance in all athletes during prolonged exercise.
6	Whether a ‘one size fits all’ or ‘indifferent (nothing really matters)’ approach to nutritional support for high performance sport is sufficient or whether athletes require a bespoke and personalized approach that can be developed according to the specific characteristics of the event and tweaked with practice and competition experience.

Abbreviation: CHO, carbohydrate.

These data indicated to her that the initial premise of the LCHF diet, to remove the need for CHO intake before or during exercise to optimize performance, could not be supported. In particular, her studies in elite Olympic-class athletes exposed to the LCHF diet for 3.5 wk showed impaired performance in carefully designed field (not laboratory) trials despite exceptionally high rates of fat oxidation in athletes eating the LCHF diet. She postulated that the higher rates of oxygen consumption associated with ATP production from fat oxidation provide another plausible mechanism for the slower speeds achieved by athletes eating the LCHF diet during exercise at the highest rates of sustainable oxygen consumption.

She also noted that race speeds continued to be impaired even when the LCHF diet was supplemented with additional fuel sources (restoration of muscle glycogen stores or the pre-exercise intake of exogenous ketone esters). This, she argued, is because adaptation to the LCHF diet downregulates muscle CHO use and prevents ingested or available CHO supplies from achieving their previous rates of oxidation, thus curtailing their capacity to increase ATP production from available oxygen supply.

Noakes maintained that equivalent performance in athletes following either dietary intervention must prove that muscle glycogen is not the obligatory fuel for human exercise performance. Instead, he contends that the only proven benefit of CHO ingestion on exercise performance, first established nearly a century ago, is through the prevention of a falling blood glucose concentration, especially during more prolonged exercise. This can be addressed by ingesting just a sufficient amount of CHO during exercise to maintain normoglycemia with sufficient glucose in the small glucose pool (SGP) in the liver and blood stream. Maintenance of normoglycemia prevents the activation of well-established centrally (brain) regulated restraint of exercise performance whenever the glucose content of the SGP begins to fall. This protective restraint is necessary to ensure that exercise terminates before glucopenic brain damage can occur. He further argued that the scientific soundness of Burke’s otherwise exceptional race walker studies is compromised by the logistical difficulties of conducting reliable RCTs with Olympic-class athletes during their preparations for international competitions. He argued that these studies establish the early (acute) metabolic and performance adaptations of elite athletes to the LCHF diet but do not necessarily represent the full range of these adaptations in these athletes when the dietary period is extended beyond 3.5 wk.

In her rebuttals, Burke contended that exercise metabolism and sporting competitions are far more complex than can be explained by a singular mechanism with a particular solution to optimize performance. Instead, she argued that competitions involve a range of challenges and performance limitations that can be addressed most effectively using a suite of dietary strategies that offer independent and overlapping benefits. Common events in high performance sport can be optimized by the interaction of strategies that promote high CHO availability to the brain and muscle, in which CHO provides an economical and versatile muscle fuel and both a metabolic and “sensing” (nonmetabolic) role in central nervous system activity. Meanwhile, other events in which interventions required to promote high CHO availability might produce a negative effect may benefit from adaptation to an LCHF diet to maximize fat oxidation. She agreed with the most recent evidence that even the LCHF approach to sports performance may require CHO intake during the event to achieve its best outcomes. This, she contended, proves her argument that CHO is essential for optimum human exercise performance.

Noakes concluded by noting that the topic of the debate was to review the scientific evidence for the singular effect of diet and metabolism on athletic performance, not to consider all the possible contributors to optimized athletic achievements. Finally, a list of areas for future research were identified by both authors (Table 3).

**TABLE 3:** Research agenda to resolve debate

The following studies should be conducted to contribute to a better understanding:	
1	Use of stable isotopes to measure rates of fat oxidation during exercise in subjects adapted to the LCHF diet, especially during exercise at higher intensities (>80% VO_2_max).
2	Determination of the time course of the immediate (weeks) and prolonged (months) adaptations in exercise performance in response to the LCHF diet.
3	Determination of the consequences of increased VO_2_ during exercise in athletes adapted to the LCHF diet.
4.	Comparison studies (using RCT designs) of sports performance in real world competitions in athletes adapted for the appropriate durations to the LCHF and HCLF diets. Sports could include endurance events as well as team sports.
5	Comparison of the response of elite vs “regular” athletes properly adapted to these diets for the appropriate duration to produce the optimal metabolic and physiologic adaptations.
6	Effect of optimal combinations of a suite of dietary strategies (pre-event dietary adaptation, pre-event meals, in-race intake) on sports performance.
7	Dose response to different intakes of CHO during prolonged exercise to see if prevention of hypoglycemia is the sole or most important role of CHO intake during prolonged exercise.
8	Management of beliefs (by blinding both athletes and researchers to the intervention) to maximize/standardize/remove their potential confounding effect on sports performance.

Abbreviations: CHO, carbohydrate; LCHF, low-carbohydrate high-fat; HCLF, high-carbohydrate low-fat; RCT, randomized controlled trial; VO_2_, oxygen consumption; VO_2_max, maximum aerobic capacity.

## Author contributions

Both authors read and approved the final manuscript.

## Disclaimer

This article series is designed as an Oxford-style debate. As such, participants are required to argue pro and con positions, even when that opinion may differ from their own. The views expressed in this debate do not necessarily reflect the opinion of the participants, *The American Journal of Clinical Nutrition*, or the American Society for Nutrition.

## Funding

The authors reported no funding received for this study.

## Declaration of generative AI and AI-assisted technologies in the writing process

No AI tools were used in the preparation of this paper.

## Conflict of interest

LMB has been an unpaid and short-term member of several Scientific Advisory Boards for Sports Nutrition Companies during selected periods of her career in sports nutrition: Gatorade Sports Science Institute Expert panel and Science in Sports. LMB has received travel grants from organizations related to both the carbohydrate and low-carbohydrate high-fat (LCHF) industries to present on this topic. She has worked as a sports dietitian in elite sport for 45 y, supporting high performance athletes to implement bespoke nutrition plans for their specific sporting pursuits. This includes assisting athletes to adopt periodized approaches to carbohydrate availability as well as specialized use of the LCHF diet for ultra-endurance events. TDN is the author of low-carbohydrate nutrition books and a shareholder in The Nutrition Network. TDN donates all the royalties from these activities to The Noakes Foundation. TDN’s work promotes the value of carbohydrate restricted diets for the management of specific metabolic disease states, especially those involving insulin resistance, and their potential value for athletes, not just for those participating in endurance exercise.

